# SPP1 expression serves as a potential peripheral circulating biomarker for lung cancer prognostics and drives tumorigenesis

**DOI:** 10.1016/j.gendis.2025.101994

**Published:** 2025-12-23

**Authors:** Xu-Ran Zhang, Fan-Li Sun, Bing Wei, Bing-Hua Jiang

**Affiliations:** a The Affiliated Cancer Hospital of Zhengzhou University & Henan Cancer Hospital, Zhengzhou, Henan 450008, China; b Henan Key Laboratory of Molecular Pathology, Zhengzhou, Henan 450008, China; c The Third Affiliated Hospital of Zhengzhou University, Zhengzhou, Henan 450001, China

Secreted phosphoprotein 1 (SPP1, osteopontin) is a secreted phosphorylated glycoprotein that participates in extracellular signaling, immune regulation, and epithelial–mesenchymal transition.^1^^,^^2^ A recent study published in *Nature* demonstrated that loss of SPP1 led to depletion of mesenchymal populations, whereas its overexpression drove epithelial–mesenchymal transition and tumor aggressiveness. Mechanistically, SPP1 regulated pancreatic cancer progression through "SPP1–CD61–NF-κB–BMP2/GREM1–SPP1" feedback circuit; suggesting that SPP1 and its associated molecules (CD61, NF-κB) are potential therapeutic target(s).^3^

SPP1 was indicated to be involved in lung cancer (LC) progression. However, it remains to be investigated whether SPP1 levels may serve as a circulating biomarker or affect tumor growth. In this study, we integrated multi-omics analyses, functional assays, and animal studies to demonstrate that SPP1 expression levels in peripheral blood samples may be used as a potential biomarker for LC progression and survival, and SPP1 up-regulation drives malignant cell transformation and tumor growth. The results are briefly described below.

To verify the clinical relevance of SPP1, we first examined its expression in seven pairs of LC tissues and thirty plasma samples. RNA sequencing and quantitative proteomics revealed that both SPP1 RNA and protein expression levels were most up-regulated in LC tissues compared with adjacent tissues (log_2_FC > 2, *p* < 0.01; Fig. 1A and B). Plasma samples from 30 LC patients and 24 healthy controls were analyzed by enzyme-linked immunosorbent assay. Peripheral circulating SPP1 concentrations were significantly higher in LC patients (*p* < 0.001), and its levels were strongly associated with cancer stages from stage I to stage IV (Fig. 1C). These findings were validated in different Clinical Proteomic Tumor Analysis Consortium (CPTAC) dataset, where SPP1 protein abundance was correlated with cancer stages (Normal *vs*. Stage I: *p* = 4.86 × 10^−6^; Stage I *vs*. Stage III: *p* = 3.48 × 10^−7^; Fig. 1D). Kaplan–Meier survival analysis showed that high SPP1 levels in LC patients predicted shorter overall survival (hazard ratio = 1.5, *p* = 0.04; Fig. 1E). Together, these results demonstrate that SPP1 is closely associated with LC progression and patient prognosis. Our result is a complement to a recent report that SPP1 enrichment in mesenchymal-like subpopulations was associated with epithelial–mesenchymal transition in pancreatic cancer.^3^ Notably, we showed that higher circulating SPP1 levels reflected tumor advanced stages and prognostics, highlighting its potential as a new non-invasive biomarker.

**Figure 1:**
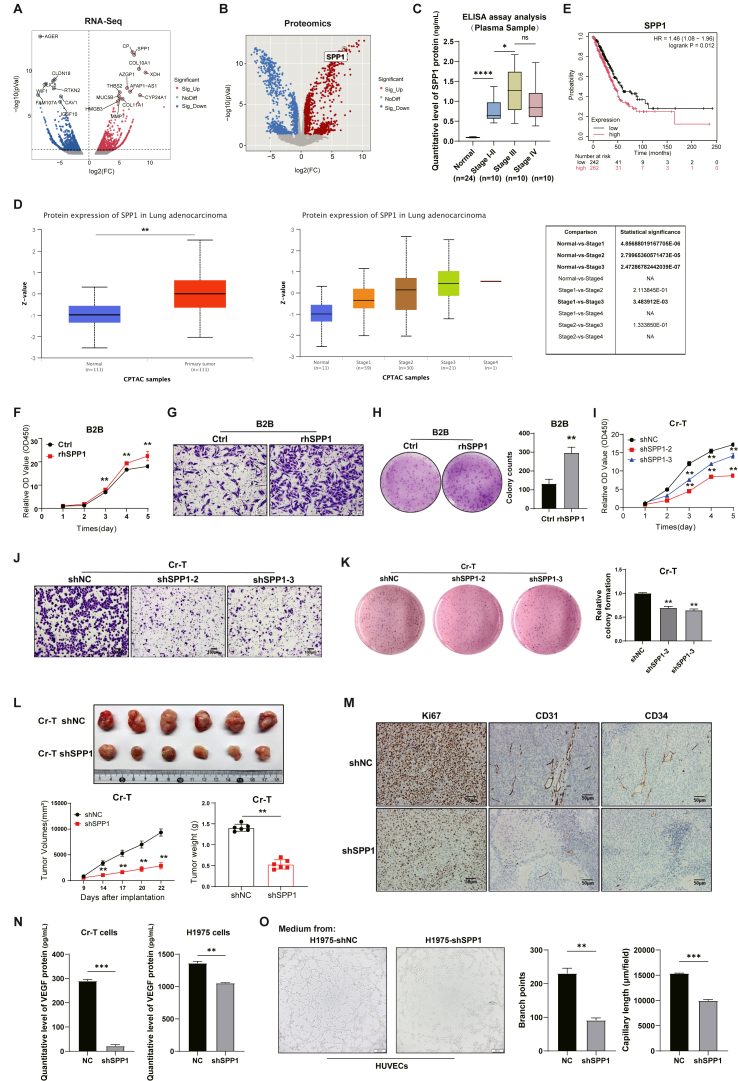
SPP1 expression and its role in lung cancer (LC) development and tumor growth. **(A, B)** RNA-sequencing analysis (A) and quantitative proteomics (B) showed that SPP1 was up-regulated in LC tissues compared with adjacent non-tumor tissues (*n* = 7, log_2_FC > 1.5). **(C)** Plasma SPP1 concentrations were measured by enzyme-linked immunosorbent assay (ELISA) in 24 healthy subjects and 30 LC patients (stages I–IV). **(D)** SPP1 protein expression levels were associated with lung adenocarcinoma (CPTAC dataset) stages. **(E)** Kaplan–Meier plot of the survival probability of LC patients with high or low SPP1 expression levels from the TCGA dataset. **(F–H)** CCK-8 assay (F), Transwell assay (G), and anchorage-independent growth assay (H) were performed using B2B cells with exogenous rhSPP1 treatment. **(I)** CCK-8 assay showed that SPP1 knockdown significantly reduced Cr-T cell proliferation rates. **(J)** Transwell assay indicated lower migration ability in Cr-T cells with SPP1 knockdown. **(K)** Anchorage-independent growth assay was performed using the Cr-T cells with SPP1 knockdown. **(L)** Xenograft tumor growth rates in nude mice were analyzed through subcutaneous injection of Cr-T shNC (control, *n* = 6) or Cr-T shSPP1 (SPP1 knockdown, *n* = 6) cells. Tumor volumes were monitored at various time periods, and final tumor weights were recorded and analyzed. **(M)** Immunohistochemical staining of CD31 and CD34 in xenograft tumors showed significantly lower microvessel densities in the shSPP1 group (scale bar = 50 μm). **(N)** ELISA measured the levels of VEGF in LC cell culture supernatants. **(O)** Tube formation assay using human umbilical vein endothelial cells (HUVECs) treated with conditioned medium from SPP1-manipulated cells. ns. not significant; ∗*p* ≤ 0.05, ∗∗*p* ≤ 0.01, ∗∗∗*p* ≤ 0.001, and ∗∗∗∗*p* ≤ 0.0001 by *t*-test.

Previous studies showed that despite being produced in numerous organs, SPP1 was expressed in only a handful of cell types at the cellular level. To investigate whether SPP1 may regulate the transformation of normal cells into cancer cells, we used human bronchial epithelial B2B cells by chronic low-dose exposure to hexavalent chromium (Cr(VI)) for six months to obtain the transformed cells (Cr-T cells) as a malignant transformation model^4^^,^^5^ (Fig. S1A). Quantitative reverse transcription PCR revealed that SPP1 transcripts were up-regulated by 10^5^-fold higher in transformed cells compared with B2B cells, and Western blotting confirmed much higher protein up-regulation (Fig. S1B and S1C), which is consistent with our above result obtained in the LC tissue and plasma samples.

Next, we investigated whether SPP1 may be sufficient to promote oncogenic phenotype, and showed that normal B2B cells treated with exogenous recombinant human SPP1 (rhSPP1) significantly enhanced cell proliferation and cell migration ability (Fig. 1F and G), especially anchorage-independent growth ability (a hallmark of malignant transformation) (Fig. 1H). To test whether SPP1 may be required for the proliferation and migration of LC cells, SPP1 was knocked down in transformed Cr-T cells (Fig. S1D). We showed that SPP1 silencing markedly reduced the transformed cell proliferation ability, migration ability, and anchorage-independent growth ability (Fig. 1I–K). We also showed high expression levels of SPP1 in the human non-small cell lung cancer cell line H1975 using quantitative reverse transcription PCR and Western blotting (Fig. S1E and S1F). Furthermore, SPP1 silencing suppressed the H1975 cell proliferation and invasion (Fig. S1G–S1I). Additionally, we showed that SPP1 knockdown inhibited p65 and BMP2 protein expression (Fig. S1J). Collectively, these results demonstrate that SPP1 expression is important in malignant transformation and may serve as a circulating biomarker for LC prognostics.

To explore the role of SPP1 *in vivo*, we conducted an animal study by implanting Cr-T cells with stable SPP1 knockdown (shSPP1) or control shRNA (shNC) into the nude mice. SPP1 silencing markedly inhibited tumor growth, decreased both tumor volume and weight by approximately 50% at 28 days post-implantation compared with control cells (Fig. 1L). Immunohistochemical staining showed that *CD31*^*+*^ and *CD34*^*+*^ microvessel densities were substantially lower in shSPP1 tumors (Fig. 1M; Fig. S1K), suggesting that SPP1 contributes to angiogenesis within the LC tumor microenvironment. In addition, we performed an enzyme-linked immunosorbent assay and found that the VEGF protein expression levels were down-regulated in the cell culture supernatants of SPP1-knockdown cells (Fig. 1N). Additionally, we conducted tube formation assays by treating human umbilical vein endothelial cells with conditioned medium from SPP1-knockdown cells or control cells. Quantitative analysis revealed that human umbilical vein endothelial cells treated with conditioned medium from the SPP1-knockdown cells inhibited tube formation (Fig. 1O). These results indicate that SPP1 not only activates tumor cells but also regulates the tumor microenvironment for inducing angiogenesis. Our findings are consistent with tumor models such as pancreatic cancer, where SPP1 loss suppressed tumor growth and metastasis. Our data thus expand the functional role of SPP1 beyond mesenchymal maintenance to include tumor growth and angiogenesis.

In conclusion, our findings demonstrate that SPP1 expression is important in malignant transformation, and circulating SPP1 levels may be a promising non-invasive biomarker for LC progression and prognosis. The stage-dependent elevation of plasma SPP1 analyzed using plasma samples (30 LC patients compared with 24 healthy controls) and CPTAC dataset correlation with tumor stages, suggests its potential for early detection of LC. Our *in vitro* functional assay and *in vivo* animal study showed that SPP1 depletion inhibited tumor growth and angiogenesis, suggesting its potential as a new therapeutic target that warrants further validation. Additional study using diverse LC subtypes, patient-derived xenograft models, and combinatorial therapeutic strategies is needed to fully evaluate its translational potential as a new therapeutic target.

## CRediT authorship contribution statement

**Xu-Ran Zhang:** Writing – original draft, Investigation, Data curation. **Fan-Li Sun:** Methodology, Investigation. **Bing Wei:** Resources, Methodology. **Bing-Hua Jiang:** Writing – review & editing, Project administration, Funding acquisition, Conceptualization.

## Ethics declaration

All human specimens utilized in this study were obtained from the Biobank of the Affiliated Cancer Hospital of Zhengzhou University, with no inclusion of patients' personal identifying information. All experiments using human samples were reviewed and approved by the Ethics Committee of the Affiliated Cancer Hospital of Zhengzhou University (Ethics Approval No. 2025-091-002). The protocol for using animal study was reviewed and approved by the Institutional Animal Care and Use Committee (IACUC) of the Affiliated Cancer Hospital of Zhengzhou University (Approval No. 2021-KY-0039-001).

## Funding

This work was supported in part by the 10.13039/501100001809National Natural Science Foundation of China (No. 82073393).

## Conflict of interests

Binghua Jiang is the member of *Genes & Diseases* Editorial Board. To minimize bias, he was excluded from all editorial decision-making related to the acceptance of this article for publication. The remaining authors declare no conflict of interests.
