# Supplementary material for: SPP1 expression serves as a potential peripheral circulating biomarker for lung cancer prognostics and drives tumorigenesis
Source: Genes Dis. 2025 Dec 23;13(3):101994. doi: 10.1016/j.gendis.2025.101994 (PMC12859187; doi:10.1016/j.gendis.2025.101994)
Supplement: Multimedia component 1 [file mmc1.docx]

**Supplementary Material**

**Materials and methods**

**Clinical samples**Lung cancer and matched adjacent non-tumor tissues (n =7) were obtained from the Biobank of the Affiliated Cancer Hospital of Zhengzhou University (Zhengzhou, Henan, China). Plasma samples were also collected from 30 patients with lung cancer and 24 healthy individuals. All samples were de-identified, and no patient information such as name, gender, or age was accessible. Portions of each specimen were fixed in formalin for histological evaluation, while the remaining tissues were snap-frozen in liquid nitrogen for molecular analysis. Histological classification and TNM staging were performed by a certified pathologist blinded to experimental outcomes using the CoPath Anatomic Pathology system.

**Table S1. Clinical and Demographic Characteristics of Lung Cancer Patients**

| **Variable** | **Lung Cancer Patients**  **(n=30)** | **Notes** |
| --- | --- | --- |
| Age, mean ± SD (range), years | 61.4 ± 12.3 (23–78) | Continuous variable presented as mean ± standard deviation (range) |
| Sex | Male: 19 (63.3%) Female: 11 (36.7%) |  |
| Smoking history | Ever smoked: 10 (33.3%) Never smoked: 20 (66.7%) | “Ever smoked” defined as patients with smoking history marked “Yes”; “Never smoked” as blank records (smoking < 100 cigarettes/lifetime) |
| TNM stage  (8th edition) | Stage I: 8 (26.7%)  Stage II: 2 (6.7%)  Stage III: 8 (26.7%)  Stage IV: 12 (40.0%) | Based on the 8th edition of AJCC TNM Staging System for Lung Cancer |
| Lung cancer subtype | Adenocarcinoma: 20 (66.7%)  Squamous cell carcinoma: 10 (33.3%) | Classified according to the 2021 WHO Classification of Lung Tumors ; both subtypes belong to NSCLC |

**RNA-Seq**

Total RNA from lung cancer tissues was isolated using the TRIzol reagent. The mRNA was subsequently captured with the VAHTS mRNA Capture Beads Kit (Vazyme, 401-01/02) following the manufacturer’s protocol. To eliminate residual genomic DNA and RNase contamination, DNase I (NEB, M0303) and RNaseOUT (Invitrogen, 10777019) were applied. Fragmentation of purified mRNA was carried out using RNA Fragmentation Reagents (Ambion, AM8740), followed by purification with Magnetic RNA Clean Beads (Vazyme, N412). The concentration and purity of mRNA were determined using a Qubit fluorometer. mRNA sequencing libraries were prepared using the KAPA Stranded mRNA-Seq Kit (KK8429) following the manufacturer’s protocol. Briefly, first- and second-strand cDNA were synthesized and the resulting double-stranded cDNA was purified. Subsequent steps included 3′ adenylation, adapter ligation, purification of ligation products, PCR amplification of the cDNA library, and final purification of the amplified library. Transcriptome sequencing was performed on the Illumina NovaSeq 6000 platform at the Department of Molecular Pathology, Henan Cancer Hospital.

**Proteomic analysis**Total proteins were extracted from lung cancer tissues using RIPA lysis buffer, followed by digestion with a 10K ultrafiltration device based on the FASP method. Peptide samples were analyzed by mass spectrometry using an Eksigent NanoLC 400 liquid chromatography system coupled to an AB Sciex TripleTOF 6600 mass spectrometer. The human reference proteome was retrieved from the UniProt database. SWATH-MS data were processed and searched against the database using DIA-NN (version 1.8.1) to identify and quantify peptides and corresponding proteins. Differentially expressed proteins were determined from three biological replicates per sample using thresholds of fold change >1.5 or <0.67 and *p* < 0.05.

**ELISA assay**

Plasma SPP1 levels were measured by using a human SPP1 ELISA kit (FineTest, No. ER1214), and VEGF levels in LC cell culture supernatants were measured by using a human VEGF ELISA kit (HUABIO, No. EH0075) following the manufacturer’s instructions. Briefly, 100 μL of standards or samples were added to each well and incubated at 37 °C for 90 min. After washing twice, 100 μL of biotin-conjugated antibody working solution was added and incubated for 60 min at 37 °C. Plates were then washed three times, followed by incubation with 100 μL of HRP–streptavidin working solution for 30 min at 37 °C. After five additional washes, 90 μL of TMB substrate solution was added and incubated for 10–20 min at 37 °C in the dark. The reaction was terminated by adding 50 μL of stop solution, and absorbance was immediately measured at 450 nm using a microplate reader. The standards provided in the kit were analyzed in parallel to generate a standard calibration curve. The mean OD₄₅₀ value of each sample (measured in duplicate) was corrected by subtracting the blank well reading, and the plasma SPP1 levels and VEGF concentrations in cell culture supernatants were calculated according to the standard curve.

**Cell culture**

The normal human bronchial epithelial cell line B2B and NSCLC cell line H1975 were purchased from the American Type Culture Collection (ATCC, USA). The Cr (VI)-transformed cell line (Cr-T) was established by continuous exposure of B2B cells to 1 μM potassium dichromate for 6 months. Cells were cultured in DMEM supplemented with 10% fetal bovine serum (FBS) and maintained at 37 °C in a humidified atmosphere containing 5% CO₂.

**RNA extraction and quantitative real-time PCR analysis**

Total RNA was isolated from the indicated cells using TRIzol reagent (Invitrogen, Waltham, MA, USA) following the manufacturer’s instructions. Complementary DNA (cDNA) was synthesized from 1 μg of total RNA using the HiScript III All-in-One RT SuperMix for qPCR (Vazyme, Nanjing, China). mRNA expression levels were quantified by real-time PCR, with GAPDH serving as the internal reference. The sequences of primers and shRNAs used in this study are listed below.

| **Genes** | **Sequence** |
| --- | --- |
| SPP1-Forward Primer | CTCCATTGACTCGAACGACTC |
| SPP1-Reverse Primer | CAGGTCTGCGAAACTTCTTAGAT |
| GAPDH-Forward Primer | GACAGTCAGCCGCATTCTTCT |
| GAPDH-Reverse Primer | GCGCCCAATACGACCAAATC |

| **Genes** | **Sequence** |
| --- | --- |
| shSPP1-2-Forward | CCGGCCACAAGCAGTCCAGATTATACTCGAGTATAATCTGGACTGCTTGTGGTTTTT |
| shSPP1-2-Reverse | AATTAAAAACCACAAGCAGTCCAGATTATACTCGAGTATAATCTGGACTGCTTGTGG |
| shSPP1-3-Forward | CCGGCTTTACAACAAATACCCAGATCTCGAGATCTGGGTATTTGTTGTAAAGTTTTT |
| shSPP1-3-Reverse | AATTAAAAACTTTACAACAAATACCCAGATCTCGAGATCTGGGTATTTGTTGTAAAG |

**Western blot**

Cells were lysed in ice-cold buffer supplemented with PMSF protease inhibitor (Beyotime, Jiangsu, China). Equal amounts of protein were separated by SDS–PAGE on 8% polyacrylamide gels and transferred onto PVDF membranes (Thermo Scientific, Waltham, MA, USA). Membranes were blocked with 5% skim milk for 2 h at room temperature and incubated with primary antibodies overnight at 4 °C. After washing, membranes were incubated with HRP-conjugated secondary antibodies, and signals were detected using an ECL reagent and imaged with a chemiluminescence detection system (Thermo Scientific, USA). The primary antibodies used in this study included anti-SPP1 (Bio-Worlde, BS1264) , anti-CD61 (HUABIO, SJ19-09), anti -p65 (BIOWORLD, BS1253), anti-BMP2 (HUABIO, JE10-29) and anti-GAPDH (Abways, AB0036).

**Cell proliferation and migration assay**

Cell proliferation was assessed using the Cell Counting Kit-8 (CCK-8; Vazyme, Nanjing, China) according to the manufacturer’s instructions. Cells were seeded in 96-well plates and incubated at 37 °C. Optical density (OD) at 450 nm was measured at 24, 48, 72, 96, and 120 h after seeding using a microplate reader.
 For the migration assay, 150 μL of serum-free medium containing 5 × 10⁴ cells was added to the upper chamber, while 600 μL of complete medium with 10% FBS was placed in the lower chamber. After incubation for 18–24 h, cells were fixed and stained with crystal violet for 15 min. Non-migrated cells were removed, and migrated cells on the lower membrane surface were observed under a light microscope.

**Three-dimensional (3D) Soft Agar Colony Formation Assay**

The 0.8% and 1.2% agarose gels were prepared by dissolving 0.24 g agarose in 30 mL and 20 mL phosphate-buffered saline (PBS), respectively, followed by autoclaving and maintaining at 60°C to prevent solidification. For the bottom layer, 1.2% agarose gel was mixed with an equal volume of 2×DMEM medium after heating to 100 °C, and 1 mL of the mixture was added to PBS-pre-rinsed 6-well plates, which was allowed to solidify at room temperature for 30 min. For the top layer, 0.8% agarose gel was equilibrated at 42 °C, then mixed with an equal volume of 2×DMEM medium containing target cells (seeded at 2000 cells per well). Two milliliters of the cell-agarose mixture were added to each well with solidified bottom gel, and the plates were incubated at 37 °C after solidification at room temperature for 1 h. Five hundred microliters of medium were added the next day, and the medium was replenished every 3 days to avoid desiccation. After 3 weeks of culture or when visible colonies formed, cells were stained overnight with 0.5% nitroblue tetrazolium (NBT) solution. The stain was removed, wells were washed, and colonies were photographed and counted.

**Mouse xenograft model**

Twelve female NOD-SCID mice (3–4 weeks old) were randomly divided into two groups (n = 6 per group) and maintained under specific pathogen-free (SPF) conditions.

Cr-T cells stably expressing shSPP1 or control shRNA (shNC) were harvested, resuspended in serum-free medium, and subcutaneously injected into both flanks of each mouse (5 × 10⁶ cells in 100 μL per injection). Tumor dimensions were measured every three days once tumors reached approximately 6 × 6 mm, and growth curves were generated from 5–7 consecutive measurements. After 21 days, mice were euthanized, and subcutaneous tumors were excised, photographed, and weighed. Tumors were fixed in formalin, embedded in paraffin, sectioned, and subjected to immunohistochemical staining for Ki67, CD31, and CD34.

**Statistical Analysis**

Differential expression screening for RNA-seq and quantitative proteomic data of LC tissues was performed using the criteria of |log2 fold change (log2FC)| ≥ 1.5 and p-value < 0.05. For plasma ELISA assays, two-tailed unpaired Student’s t-test was used to compare differences between two groups. The correlations between SPP1 expression levels and overall survival (OS) of LC patients were analyzed using the Kaplan-Meier Plotter (https://kmplot.com/analysis/). Differences in SPP1 expression levels across different stages of LC tissues were statistically evaluated via the built-in statistical module from the UALCAN public database (https://ualcan.path.uab.edu/cgi-bin/CPTAC-Result.pl?genenam=spp1&ctype=LUAD). For data processing of *in vitro* and *in vivo* experiments, additional data collation and visualization were conducted by using GraphPad Prism 8.0 (GraphPad Software Inc, San Diego, CA, USA), and two-tailed unpaired Student’s t-test was applied to compare differences between two groups. All data were presented in line with database-specific output standards, and the results were considered significantly different at p-value < 0.05.

**Supplementary figures**


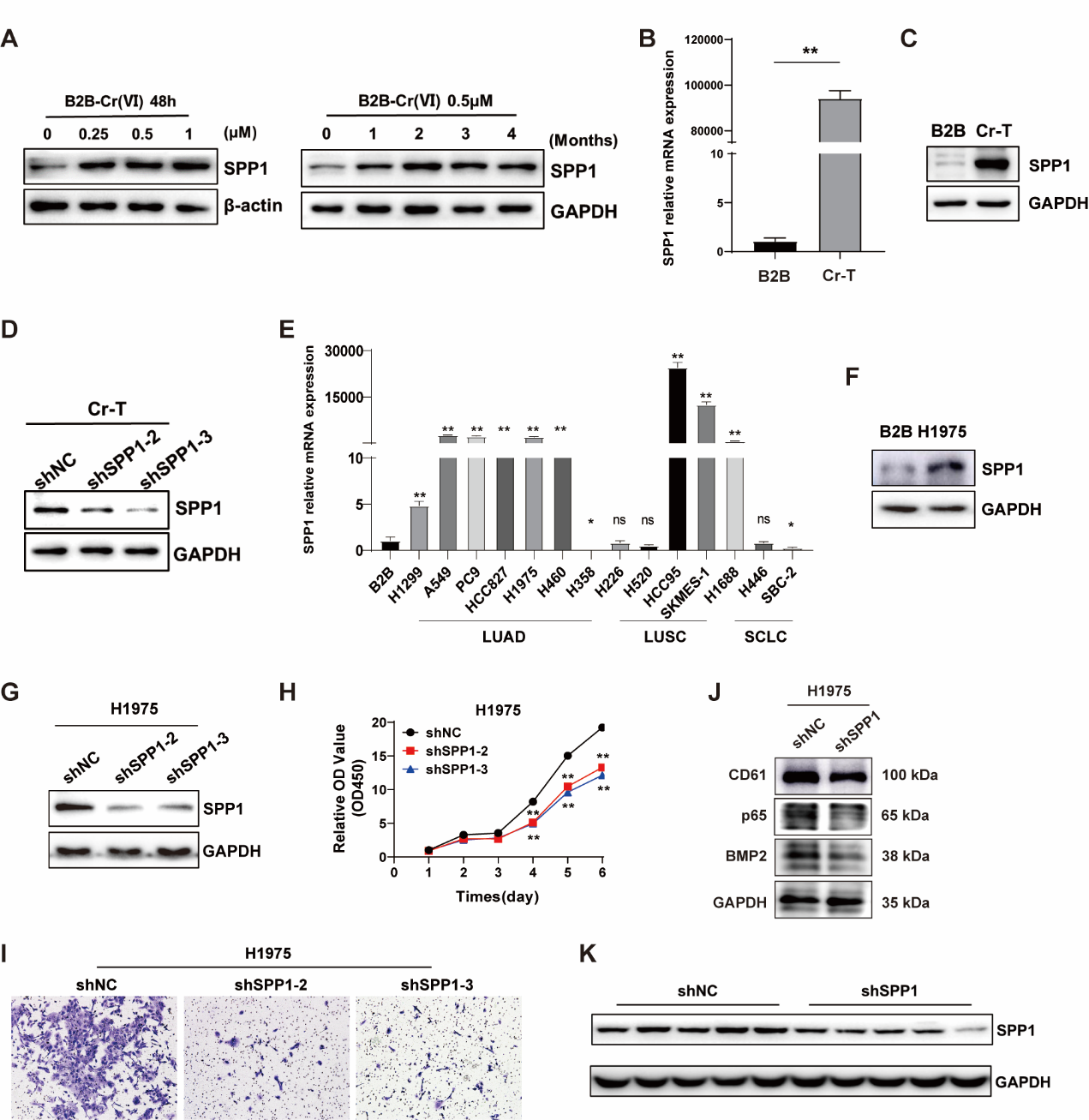


**Figure S1** SPP1 expression levels and its role in lung cancer development. **(A)** Western blotting showed SPP1 expression levels were upregulated in B2B cells with increasing Cr(IV) treatment time. **(B)** RT-qPCR and **(C)** Western blotting showed SPP1 expression levels were much higher in Cr-T cells compared to B2B cells. **(D)** Western blotting showed the expression levels of SPP1 in Cr-T cells. **(E)** RT-qPCR showed that SPP1 expression levels were much higher in LC cells compared to normal cells. Western blotting showed the SPP1 expression levels in **(F)** H1975 cells, B2B cells and **(G)** in H1975 cells with or without SPP1 knockdown. **(H)** CCK-8 assay showed that SPP1 knockdown significantly reduced H1975 cell proliferation rates. **(I)** Transwell assay indicated lower migration ability of H1975 cells with SPP1 knockdown. **(J)** Western blotting showed that p65 and BMP2 expression levels were downregulated in SPP1 knockdown cells**. (K)**Western blotting showed the SPP1 expression levels in LC mouse tumors. ns. not significant; significant difference at *p≤0.05, or **p≤0.01 by *t* test.
